# Effects of caesarean section on maternal health in low risk nulliparous women: a prospective matched cohort study in Shanghai, China

**DOI:** 10.1186/1471-2393-10-78

**Published:** 2010-12-02

**Authors:** Bing-shun Wang, Li-feng Zhou, David Coulter, Hong Liang, Ye Zhong, Yu-na Guo, Li-ping Zhu, Xiao-ling Gao, Wei Yuan, Er-sheng Gao

**Affiliations:** 1 Department of Biostatistics, Shanghai Jiao Tong University School of Medicine, 227 South Chongqing Road, Shanghai 200025, PR China; 2 Planning and Funding, Waitemata District Health Board, Private Bag 93-503, Takapuna, North Shore 0740, New Zealand; 3 264 Highgate, Roslyn, Dunedin 9010, New Zealand; 4 Department of Epidemiology and Social Sciences, Shanghai Institute of Planned Parenthood Research, 2140 Xie Tu Road, Shanghai 200032, PR China; 5 Center for Clinical Epidemiology and Biostatistics, University of Pennsylvania, 507 Blockley Hall, 423 Guardian Drive, Philadelphia, PA 19104-6021, USA; 6 International Peace Maternity and Child Health Hospital, Shanghai Jiao Tong University School of Medicine, 910 Heng Shan Road, Shanghai 200030, PR China; 7 Shanghai Women's Health Institute, 122 South Shan Xi Road, Shanghai 200040, PR China

## Abstract

**Background:**

Rates of caesarean section are progressively increasing in many parts of the world. As a result of psychosocial factors there has been an increasing tendency for pregnant women without justifiable medical indications for caesarean section to ask for this procedure in China. A critical examination of this issue in relation to maternal outcomes is important. At present there are no clinical trials to help assess the risks and benefits of caesarean section in low risk women. To fill the gap left by trials, this indication-matched cohort study was carried out to examine prospectively the outcomes of caesarean section on women with no absolute obstetric indication compared with similar women who had vaginal delivery.

**Methods:**

An indication-matched cohort study was undertaken to compare maternal outcomes following caesarean section with those undergoing vaginal delivery, in which the two groups were matched for non-absolute indications. 301 nulliparous women with caesarean section were matched successfully with 301 women who delivered vaginally in the Maternal and Children's Hospitals (MCHs) in Shanghai, China. Logistic regression model or binomial regression model was used to estimate the relative risk (RR) directly. Adjusted RRs were calculated adjusting for propensity score and medical indications.

**Results:**

The incidence of total complications was 2.2 times higher in the caesarean section group during hospitalization post-partum, compared with the vaginal delivery group (RR = 2.2; 95% CI: 1.1-4.4). The risk of haemorrhage from the start of labour until 2 hours post-partum was significantly higher in the caesarean group (RR = 5.6; 95% CI: 1.2-26.9). The risk of chronic abdominal pain was significantly higher for the caesarean section group (RR = 3.6; 95% CI: 1.2-10.9) than for the vaginal delivery group within 12 months post-partum. The two groups had similar incidences of anaemia and complicating infections such as wound complications or urinary tract infection.

**Conclusions:**

In nulliparous women who were at low risk, caesarean section was associated with a higher rate of post-partum morbidity. Those requesting the surgical procedure with no conventional medical indication, should be advised of the potential risks.

## Background

Rates of caesarean section are progressively increasing in many parts of the world, particularly among developing countries such as China [1-4]. In many Chinese hospitals, the caesarean section rate was more than 40%, while in some cases, it was up to 80% [2-4], which was much higher than the acceptable caesarean rate (5-15%) in WHO's guidelines [5]. Although the rate of caesarean section resulting in the best outcome for mothers and children continues to be a matter of debate, it is evident that a better outcome (including lower morbidity and mortality) does not necessarily result from a higher rate of caesarean sections.

In recent years, there has been an increasing tendency for pregnant women without obstetric indications for caesarean section to ask for this procedure because they perceive it to be safe and more convenient than vaginal delivery [4,6]. This situation has become a significant factor leading to the increased rate of caesarean section in China [4,6]. Currently there is much debate as to whether this surgical procedure should be performed for women without clear clinically acceptable indications [7-11]. The focus of the debate is whether caesarean section has greater benefits than vaginal delivery and is an acceptable alternative to vaginal delivery in low risk women.

Some studies were against and some were for caesarean section [12-18]. Most previous studies were retrospective and could not identify detailed significant baseline differences between the two groups of caesarean section and vaginal delivery. Because of this, it is difficult to evaluate whether the short- and long-term abnormalities (e.g. post partum haemorrhage and chronic abdominal pain) were directly due to the caesarean sections or to underlying conditions. All-cause caesareans might comprise women who need a life saving surgical intervention for the mother or baby as well as women whose need for the procedure was not clinically justified. The interpretation of these crude caesarean rates is therefore difficult. Randomized clinical trials may be a good choice to address the issue, but this is not feasible in most cases. A Cochrane Review that focused on this subject found no trials to help assess the risks and benefits of caesarean section when undertaken without a conventional medical indication. The authors of the review strongly recommended alternative research methods to gather data on the outcomes associated with different ways of giving birth [19]. It is our contention that a prospective parallel-group observational study matching or controlling for possible confounders could overcome these deficiencies in the trials. We therefore undertook a study with an indication-matched cohort design to compare the medical outcomes of mothers who had caesarean section with those who delivered vaginally. This study focused on nulliparous pregnant women who were relatively healthy, did not have a history of any serious diseases and had no serious complications during pregnancy. In other words, these women were at low risk of complications at delivery.

## Methods

**Figure 1:**
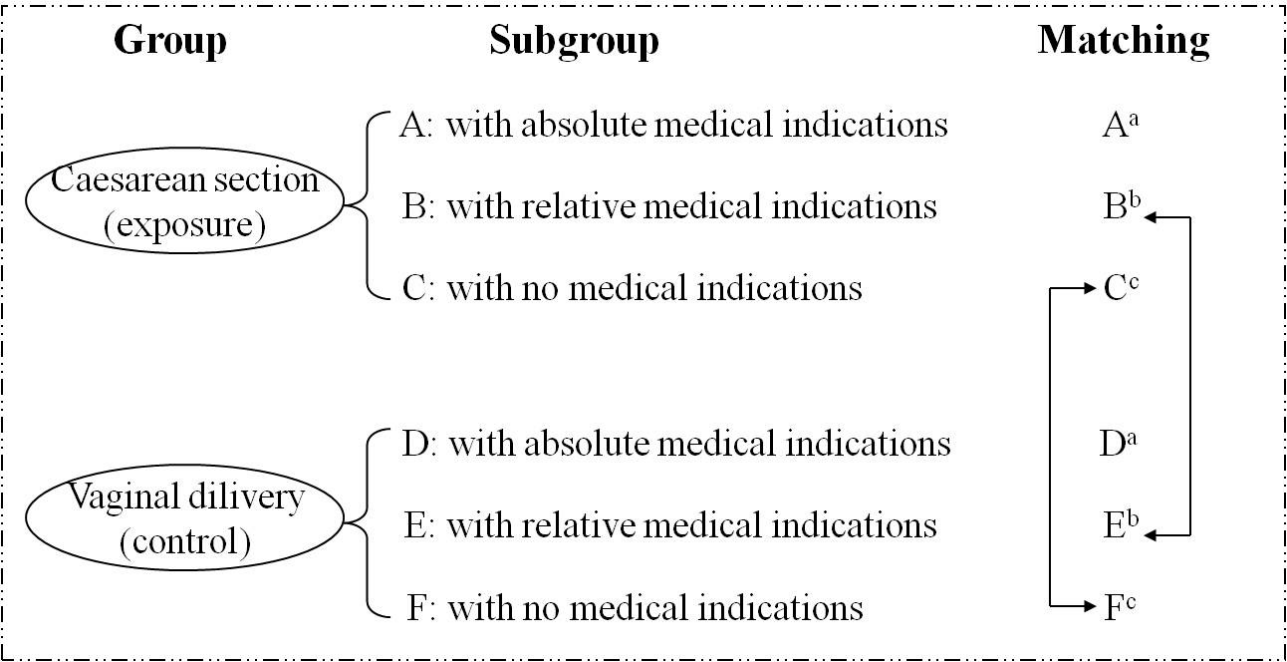
**Group classification and matching**. *^a ^Women with absolute medical indications for caesarean section were excluded in the study; ^b ^Women with relative indications in the caesarean section group were matched with those who delivered vaginally; ^c ^Women with no indications in the caesarean section group were matched with those who delivered vaginally*.

This study was an indication-matched cohort study, comparing women who had caesarean section with a comparable low risk group of women who had vaginal delivery (Figure 1). It involved pregnant women without obstetric indications (subgroup C and F in Figure 1) or with relative medical indications (subgroup B and E in Figure 1) for caesarean section, in other words low risk pregnant women. Women with absolute medical indications for caesarean section were excluded (subgroup A and D in Figure 1). There are different classification systems recommended for use in high or low caesarean delivery rate settings [20]. The absolute and relative indications for caesarean section in this study, which are shown in Table 1, were classified according to the *Practice Guidelines for Gynaecology and Obstetrics in Shanghai *[21] and the opinions of the expert team on this project.

**Table 1:** Classification of the indications for caesarean section *

Absolute indications	Relative indications
• Serious pelvic stenosis or abnormal pelvis • Severe antepartum bleeding, such as placental praevia and placental abruption • Abnormality of soft birth canal, such as scar tissue or pelvic tumour impeding presentation descent and diaphragm of vagina • Scar of uterus • Abnormal foetal presentations, such as mixed breech presentations • Pre-rupture of uterus • Foetal malformation, such as conjoined twins • After repair operation of reproductive organ fistula	• Relative cephalopelvic disproportion, such as poor engagement of foetal head and foetal macrosomia • Foetal distress • Pregnancy complications, such as cardiac disease, renal disease and liver disease in pregnancy • Other indications, such as uterine inertia

* The categories of indications were set down according to the *Practice Guidelines for Gynaecology and Obstetrics in Shanghai *[21] and the opinions of the expert team on this project. Accordingly, foetal distress refers to the presence of meconium in the amniotic fluid concomitant with increased or decreased foetal heart rate. Lactic acidosis assessment is not generally available.

### Patient selection

This study was undertaken in three district level Maternal and Children's Hospitals (MCHs) in Shanghai. The inclusion criteria were: 1) older than 20 and younger than 35 years; 2) more than 37 weeks gestation at delivery; 3) nulliparous; 4) no history of induced abortion (including medical abortion and surgical abortion); 5) no history of heart, liver, lung, kidney, endocrine or psychiatric diseases resulting in hospitalization; 6) planning to have the delivery at the present MCH and planning to live in Shanghai after delivery. The exclusion criteria were: 1) unmarried, divorced or widowed; 2) a history of spontaneous abortion; 3) multiple foetus; 4) more than 42 weeks gestation at delivery; 5) low birth weight (less than 2500 g); 6) the presence of absolute indications for caesarean section.

Nulliparous women were selected because of the 'One-Child' family planning policy in China. Moreover, this restriction should avoid possible confounding by parity in the analysis.

Women meeting these inclusion and exclusion criteria were enrolled in this study, and women with relative indications or without any obstetric indication for caesarean section were finally accepted following preliminary screening in the antenatal clinics and re-screening in the maternity wards after birth. The exposure group of eligible women included those with no identified risk and some with relative indications for caesarean section. The control group included some who had relative indications, but did not proceed to caesarean section.

The research team took no part in the clinical care of the women and did not participate in the decision to have a caesarean section.

The women in the caesarean section group were matched with those who delivered vaginally (Figure 1). The matching criteria were:

1. The absence of any indication for caesarean section (subgroup C and F in Figure 1).

2. The presence of a relative indication (subgroup B and E in Figure 1). Where possible the women were matched by the precise condition. If there could not be an exact match a similar indication was accepted, or the pairs were matched according to the presence of any relative indication.

3. A delivery date within 20 days of each other. The subject delivering first received precedence for matching.

4. Delivery in the same hospital.

### Data collection

**Figure 2:**
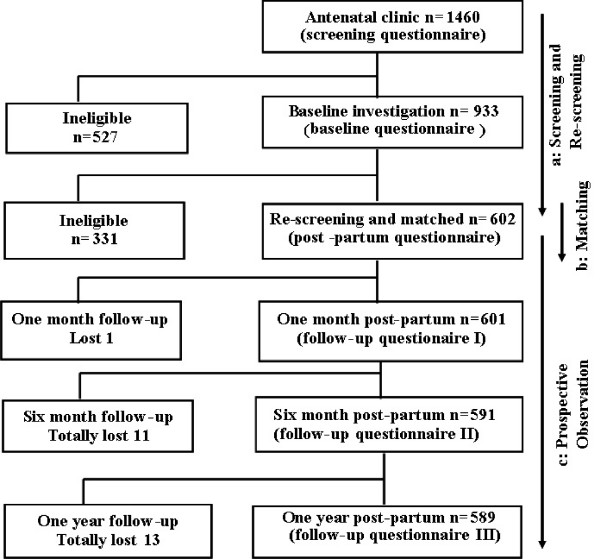
**Flow diagram of screening, matching and follow-up indicating losses from the original cohort of the study**. *^a ^The preliminary screening was conducted in the antenatal clinics and re-screening in the maternity wards after birth; ^b ^Eligible women in the caesarean section group were matched with those who delivered vaginally (Figure 1) according to the matching criteria; ^c ^Post-partum investigations were made in the maternity wards, and follow-up of 1 month, 6 months and 12 months post-partum in the Maternity and Child Health Institutes affiliated to MCHs*.

Information collected was recorded on questionnaires (see Additional file 1). A baseline questionnaire was completed at the time of preliminary screening in the antenatal clinics to determine preliminary eligibility for the study (Figure 2). Details recorded were demographic characteristics, smoking habits, alcohol use and medical history. In addition, the weight and height before pregnancy and health events and medicine use during pregnancy were recorded. A post-partum questionnaire was completed by interview in conjunction with the obstetrical record. The data recorded were method of delivery, gestational weeks at delivery, medical events during delivery, and the occurrence of complications post-partum. The volume of blood loss was recorded from the start of labour until two hours post-partum by a measurement cup (when losing a large amount of blood) and weighing gauzes. Post-partum haemorrhage (PPH) was defined as the total loss of blood of more than 400 ml from the start of labour until 2 hours post-partum, according to the definition suggested by the Chinese Collaboration Group of Bleeding Post-partum [22]. This definition of PPH was different from the conventional one of blood loss of more than 500 ml from the genital tract within 24 hours of delivery. However the definition used in this study was considered more practical and easier to operate by the health professionals in the MCHs and similar definitions were also used in other clinical investigations with a focus on PPH [23-25].

Follow-up interviews were conducted at 1 month, 6 months and 12 months post-partum. Outcomes recorded were infection (reproductive tract infection, urinary infection and wound complications), anaemia (mainly iron-deficiency anaemia with haemoglobin lower than 110 g/L post-partum), puerperal fever, chronic abdominal pain and rehospitalization. The temperature was taken at least four times daily and puerperal fever was defined as a temperature equal to or greater than 38°C occurring at least twice within the first 10 post-partum days, exclusive of the first 24 hours. Chronic abdominal pain was defined as non-cyclic pain in the lower abdomen for at least 4 months, interfering with daily activities.

All follow-up interviews were completed by trained health staff in the MCHs where the women were enrolled. The interviewers were blind to the method of delivery in the follow-up interviews at 1, 6 and 12 months post partum. A clinician in each MCH was responsible for collecting the data and completed the questionnaires with the help of experienced midwives or health workers. Because of concern for misclassification of medical indications at the matching stage in the study, a quality control procedure was used, guided by the senior doctors and audited by a member of the expert team, to ensure the fundamental 'eligibility' matching criteria were met. The collection of follow-up data was completed by March 2004.

### Routine practice

The MCHs in this study were government hospitals. They were similar in obstetric practice which mainly followed the official guidelines on clinical practice [2,21]. Routine active management was given by medical care personnel to all women. Analgesia such as epidural analgesia would be offered if indicated or women requested it. The caesarean sections and operative vaginal deliveries were undertaken by experienced obstetricians. Women having a caesarean section or those at high risk of infection should be offered prophylactic antibiotics, such as a single dose of first-generation cephalosporin (and metronidazole if necessary), to reduce the risk of post-operative infections. Active management included the following aimed at preventing PPH: routine intramuscular (i.m.) injection of a preventive single dose of oxytocin with delivery of the baby, controlled cord traction and uterine massage after delivery of the placenta. The preventive single i.m. dose of oxytocin may be 10 or 20 IU. This varied in different hospitals, but is the same for caesarean section and vaginal delivery in the same hospital. In addition to the routine single i.m. dose of oxytocin, extra doses of oxytocin by intravenous continuous drip or other uterotonics (such as carbetocin, prostaglandin PGE_2a _or carboprost) may be administered after delivery. This decision relied on the clinicians' assessment (e.g. atonic uterus). Administration of all uterotonics and relevant details of active management of labour were recorded.

### Sample size

We estimated the overall maternal morbidity would be 8% of the caesarean section group [26,27]. Thus, an equally divided sample of 540 was deemed sufficient for the detection of a risk ratio of 5.0 compared with the vaginal delivery group, with a type I error (two-sided) of 5% and a power of 90%. On the assumption of a 10% rate of loss to follow-up, we established an overall target sample size of 600.

### Statistical methods

As this was a matched prospective comparative study, a stratification strategy was used for examining the matched data [28,29]. Previous studies have indicated that a number of factors contribute to caesarean section [2-4]. Data collected in the current study indicated that the factors related to caesarean section did not distribute in good balance. It was therefore decided to use a propensity score technique [30,31] in the analysis to balance the differences of these factors between the two study groups. The covariates were average monthly income per capita, body mass index (BMI), maternal confidence in vaginal delivery, preference for caesarean section, prepartum self-rating depression scale (SDS), birth weight and doula support, which were combined to yield a propensity score through stepwise logistic regression (data not shown).

Comparisons between groups employed t-test or Wilcoxon rank sum test for continuous variables, Pearson Chi-square for categorical data, and Cochrane-Mantel-Haenszel Chi-square for ordered categorical data. When the incidence was smaller than 10%, a logistic regression model was employed which used odds ratio (OR) to estimate relative risk (RR); while for a more common condition, a binomial regression model was used to estimate the RR directly. Relative risks were calculated adjusting for propensity score and medical indications. The routine clinical practice that was accompanied by the method of delivery was not adjusted additionally.

Epi Data and SAS software version 8.2 (SAS Institute, Cary, NC, USA) were employed for data management and statistical analysis in this study.

### Ethical issues

Approval from the local research ethics committee in the Shanghai Institute Of Planned Parenthood Research/WHO Collaborating Centre for Research in Human Reproduction was granted for the study protocol on 10 January 2001 (reference code: 20010101), and the study was also accepted by the heads of the hospitals involved. Individual informed consent was obtained during the initial screening.

## Results

A total of 1460 pregnant women were screened for eligibility at the antenatal clinics between 2001 and 2003. There were 933 women who met the inclusion criteria and had no exclusion criteria. Of these, 301 had caesarean section and were matched successfully with 301 women who delivered vaginally.

A total of 13 cases (2.2%) were lost to follow-up (Figure 2), 6 (2.0%) in the caesarean section group and 7 (2.3%) in the vaginal delivery group. The proportions and reasons for loss of follow-up were similar in the exposure and control groups. In addition, the characteristics at baseline were also similar in women followed up and those lost to follow-up in both the exposure and control groups.

### Demographic characteristics of participants

The average age of women in both groups was about 25 years (Table 2). Women in both groups had a similar distribution of education level, and years of marriage (see Additional file 2: Supplementary Table S1). A majority of the subjects had an education level of high school or higher, i.e. 265 (88.0%) in the caesarean section group and 251 (83.4%) in the vaginal delivery group. More than half of the women in both groups were workers and employees in factories and companies (53.2% in caesarean section group and 54.5% in vaginal delivery group). The caesarean section group tended to have more with a professional occupation and a higher income level, but the differences were not statistically significant (see Additional file 2: Supplementary Table S1). There was one smoker in the vaginal delivery group and none in the caesarean section group and 4 and 3 respectively drank alcohol.

**Table 2:** Maternal Characteristics by mode of delivery

Characteristic	Caesarean section (n = 301) N (%)	Vaginal delivery (n = 301) N (%)
Age (year) (Mean ± SD)	25.4 ± 2.7	24.9 ± 2.6
Body mass index before pregnancy (Mean ± SD)	20.4 ± 2.7	20.0 ± 2.5
Significant medical history 1 year before pregnancy	18 (6.0)	24 (8.0)
Significant medical history during pregnancy	23 (7.6)	25 (8.3)
RTI during pregnancy*	16 (5.3)	11 (3.7)
No indications for caesarean section	127 (42.2)	127 (42.2)
Relative indications for caesarean		
Foetal distress	104 (59.8)	117 (67.2)
Cephalopelvic disproportion	49 (28.2)	46 (26.4)
Other indications	21 (12.1)	11 (6.3)

* RTI: Reproductive tract infection.

### Medical history

No significant differences were found between the two groups in medical history during the year before pregnancy, nor during pregnancy (Table 2). There was one woman in each of the two groups who suffered preeclampsia. The incidence of threatened abortion was respectively 14.4% and 17.7% in the caesarean section and vaginal delivery groups (χ^2 ^= 1.20, *P *= 0.27).

### Obstetric details

Women in both groups had a similar duration of gestation when delivered (χ^2 ^= 0.46, *P *= 0.64), with most at 39 weeks. In the vaginal delivery group, 215 (71.4%) delivered spontaneously, 86 (28.6%) needed forceps assistance and 297 (98.7%) women had mediolateral episiotomies. In the caesarean section group, there were 134 (44.5%) women who had elective caesarean section and 167 (55.5%) who had caesarean section decided during labour. Women in both delivery groups had a similar distribution of relative indications for caesarean section as listed in Table 2. The proportion of relative cephalopelvic disproportion was respectively 16% and 15% in the caesarean section and vaginal delivery groups. Among those with relative indications, foetal distress ranked first in both groups. Epidural analgesia or combined spinal-epidural analgesia was administrated for 141 (46.8%) in the caesarean section group and 125 (41.5%) in the vaginal group respectively for labour and delivery. Prophylactic antibiotics were used for all in the caesarean section group and 145 (48.2%) in the vaginal delivery group. In addition to the routine single i.m. dose of oxytocin administered at delivery, extra uterotonics were given after delivery for 277 (95.9%) in the caesarean section group and 230 (76.7%) in vaginal delivery group (see Additional file 2: Supplementary Table S2).

### Post-partum morbidity during hospitalization

Complications during post-partum hospitalization were mainly haemorrhage, infection and fever. The incidence of total complications was 2.2 times higher in the caesarean section group (Table 3).

**Table 3:** Incidence of complications of women during hospitalization post-partum by mode of delivery

Factors	Caesarean section N (%)	Vaginal delivery N (%)	RR	95%CI	aRR*	95%CI
Total complications^†^	29 (9.4)	15 (5.0)	1.9	1.1-3.5	2.2	1.1-4.4
Haemorrhage^#^	12 (4.0)	2 (0.6)	6.0	1.4-26.6	5.6	1.2-26.9
Infection^$^	7 (2.3)	3 (1.0)	2.3	0.6-9.0	2.3	0.5-9.8
Puerperal fever	13 (4.3)	10 (3.3)	1.3	0.6-2.9	1.8	0.7-4.5

* Adjusted for propensity score and medical indications.

^† ^Some patients had more than one. When fever and infection co-existed in a woman, it was counted only once in the total morbidity statistics.

^# ^Post-partum haemorrhage was defined as the total loss of blood more than 400 ml from the start of the labour until 2 hours post-partum [22].

^$ ^Including endometritis, wound infection, urinary infection.

When individual complications were studied, it was found that the caesarean section group had a relative risk of 5.6 for post-partum haemorrhage compared with the control group, after adjusting for propensity score and the relative indications for caesarean section (Table 3). The medians (lower and upper quartile) of total blood loss during labour and within 2 hours post-partum were respectively 200 ml (200-300 ml) and 170 ml (110-200 ml) in the caesarean section group and control group and the difference was highly significant (Wilcoxon rank sum test, Z = 13.81, *P *< 0.0001). Because of uterine atony in four and tear of the uterine incision in one, the amount of PPH of five women was more than 1000 ml. These patients were all in the caesarean section group and were given blood transfusions.

Rates of puerperal infection or fever post-partum in hospital did not show any statistically significant differences between the two groups (Table 3). The median (q1-q3) days stayed in hospital after delivery were 8 (7-10) and 6 (5-7) for caesarean section and vaginal delivery groups respectively. Telephone interview was applied after hospital discharge.

### Morbidity after discharge and within one year post-partum

The clearing time of lochia was recorded in most cases at the first follow-up, which was the end of the first month post-partum. Most women (60%) had a clearing period of 4 to 6 weeks post-partum. Women in the caesarean section group had a longer time of clearing than the control group (χ^2^_CMH _= 6.41, *P *= 0.01).

At one month post-partum, the total incidences of all problems measured were 8.0% and 7.3% in the caesarean section group and control group respectively. Longer term or delayed problems are listed in table 4. 'Wound complications' refers to persisting incisional pain without infection plus break-down of abdominal wounds and episiotomies due to infection or abscess. The most frequent problems within one year after discharge included anaemia, reproductive tract infection, wound complications and waist/back pain. No statistically significant differences were found for these events in the two groups (Table 4).

**Table 4:** Clinical events within one year after discharge by mode of delivery #

Factor	Caesarean section N (%)	Vaginal delivery N (%)	RR	95%CI	aRR*	95%CI
Anaemia	40 (13.6)	40 (13.5)	1.0	0.7-1.5	1.2	0.7-1.8
RTI	20 (6.8)	26 (8.87)	0.8	0.4-1.3	1.0	0.5-1.8
Wound complications^†^	31 (10.5)	32(10.9)	1.0	0.6-1.5	1.2	0.7-1.9
Waist/back pain	60(20.3)	50 (17.0)	1.2	0.9-1.7	1.2	0.8-1.7
Chronic abdominal pain	13 (4.4)	5 (1.7)	2.6	0.9-7.2	3.6	1.2-11.0
Rehospitalization	5 (1.7)	5^$ ^(1.7)	1.0	0.3-3.4	0.7^‡^	0.2-3.6

# The clinical event was counted only once when women had the same outcome twice or more after discharge from hospital at 1, 6 and 12 months post-partum.

* Adjusted for propensity score and medical indications.

^† ^Wound complications refers to incisional pain without infection and delayed healing of abdominal incisions and episiotomies due to infection or abscess not manifested while in hospital, all reported at follow-up interviews.

$ There were 5 women (6 times) rehospitalized due to medical reasons within one year post-partum in the vaginal delivery group.

^‡^Exact Logistic regression.

The incidence of chronic abdominal pain was 4.4% and 1.7% respectively in the caesarean section group and vaginal delivery group (adjusted RR = 3.6, 95%CI 1.2-10.9) (Table 4). There were 5 women (6 times) rehospitalized for medical reasons within one year post-partum respectively in both groups (χ^2 ^= 1.17, *P *= 0.28). Four women were rehospitalized within one month post-partum: one due to fever and one due to mastitis in the caesarean section group, and two women due to mastitis in the vaginal delivery group. There were four women rehospitalized between one and 6 months post-partum: three women due to fever, mastitis and gallstone respectively in the caesarean section group, and one woman due to adenoma of the parathyroid in the vaginal delivery group. Three women were rehospitalized between 6 and 12 months post-partum: all three women were in the vaginal delivery group, two women due to ruptured corpus luteum cyst and one woman due to ectopic pregnancy.

## Discussion

Caesarean section is now the most frequently performed major obstetric operation in China. In some areas, caesarean delivery on maternal request accounted for half of all caesarean births [2-4,32]. Investigation of maternal medical outcomes of subjects with non-medically indicated caesarean section is therefore very important. Our indication-matched prospective cohort study should help to minimize confounding by indication and should add important insights on the effects of caesarean section on maternal health.

### Incidence of haemorrhage post-partum

It was found that the women in the caesarean section group had a relative risk of haemorrhage of 5.6 compared with the control group. Although routine active management was offered by skilled medical care personnel to all women, the number of years of clinical experience of the health workers for vaginal delivery was smaller, It was also found that more women in the caesarean section group were given extra uterotonics after delivery compared with the vaginal group (see Additional file 2: Supplementary Table S2). Both of these factors would tend to reduce PPH and thus the observed excess of PPH in the caesarean group is strengthened. Cheng reported that the incidence of haemorrhage was respectively 3.5% and 1.8% (definition of post-partum haemorrhage not specified) in two groups between 2000 and 2002 [26], and Zhu reported that the incidence was respectively 15.3% and 7.5% (using the traditional definition of haemorrhage as blood loss more than 500 ml from the genital tract within 24 hours of delivery) in two groups between 1998 and 2001 [27]. Although these numbers were all from clinical studies in Shanghai in recent years, the incidences varied. This might be due to differences in the definitions of post-partum haemorrhage and methods of assessing the volume of blood loss. However, all studies indicated an increased risk of haemorrhage in the caesarean section group.

### Incidence of infection

The common complications following caesarean section in the short term included infection after surgical operation (including endometritis, wound infection and urinary infection.) and fever. No statistically significant differences for these were found in the two groups. This is thought to be a result of improved perinatal medical practice, such as strict aseptic technique, and prophylactic use of antibiotics reducing the frequency of infection. At present clinical staff comply strictly with the new official national practice guidelines on the use of antibiotics: '*the Practice Guidelines for Standardized Use of Antibiotics' *[33]. In our study prophylactic antibiotics were used for all of the caesarean section group and nearly half in the vaginal delivery group (see Additional file 2: Supplementary Table S2). Prophylactic antibiotics can reduce the incidence of endometritis following caesarean section by two thirds to three quarters [34]. It has been reported that there was an increase in wound infection (RR = 3.5; 95% CI, 1.8-6.7) with caesarean delivery without labour compared with spontaneous vaginal delivery [12]. Another study found that women who had caesarean delivery were more likely to be rehospitalized with obstetrical surgical wound complications (RR = 30.2; 95% CI, 18.8-47.4) when compared with women who had spontaneous vaginal delivery [15]. The difference between our study and theirs may be due to different study populations and the very high proportion of Chinese women who had an episiotomy in our study.

### Incidence of chronic abdominal pain

In this study, it was found that women with caesarean section had a relative risk of 3.6 for chronic abdominal pain compared with those having vaginal delivery, confirming the finding in a Brazilian study [16]. Although the causal mechanism of chronic abdominal pain is not completely understood, the common reasons may be abdominal adhesion after surgical operations and pelvic inflammatory adhesions [35]. Women should be counselled about this when requesting caesarean section.

### Rehospitalization due to illness post-partum

Recent studies showed that rehospitalizations in the first one or two months after giving birth were more likely in planned caesarean when compared with planned vaginal births [15]. The present study found that the total incidence of rehospitalization within one year post-partum was 1.7%, and no difference was found between the two groups. This low rate might be attributed to the fact that all the participants in this study were relatively healthy women, with a low risk of complications.

### Limitations of this study

Caesarean sections and vaginal deliveries have different effects on women's health as shown in this study. The findings are consistent with the WHO global survey in Africa and Latin America in 2004-05 and in Asia in 2007-08, which is mainly an ecological study at institutional level [36,37]. However, some obstetric practices in China, such as the routine use of episiotomy during vaginal delivery, may limit the validity of comparisons with other countries where clinical practice is substantially different [2,3]. In reality, this may under-estimate the risk of caesarean section in China.

Due to the insufficient sample size, we were not able to subdivide the two study groups into categories such as spontaneous vaginal delivery, operative vaginal delivery, elective caesarean section or caesarean section decided during labour as defined by other surveys [36,37]. We were also not able to differentiate the effects of the different modes of delivery, on the risk of some uncommon complications such as maternal mortality, venous thromboembolism and hysterectomy. In addition, the conclusions of this study should not be extended to those women with absolute indications for caesarean section.

## Conclusion

Caesarean section in low risk nulliparous Chinese women carries increased risks over vaginal delivery. Those requesting caesarean section without conventional obstetric indications or medical indications for mother or foetus, should be advised of these potential risks.

## Competing interests

The authors declare that they have no competing interests.

## Authors' contributions

BW undertook the field work, completed the data analyses and draft. LZ designed and supervised the study and was the study guarantor. DC critically reviewed the draft and modified the text significantly. HL drafted the paper under supervision of LZ. YZ, LPZ and XG helped to design the study. YG assisted with managing the project. WY and EG provided expert knowledge during the design and made comments on the draft. All authors read and approved the final manuscript.

## Pre-publication history

The pre-publication history for this paper can be accessed here:

http://www.biomedcentral.com/1471-2393/10/78/prepub

## Supplementary Material

Additional file 1 **The first additional file is compressed by open source software '7-Zip' (**http://www.7-zip.org/**)**. The 'additional file 1' includes 11 separate questionnaires, which were originally written in Chinese for the study. They were converted from the original WORD version to 'Adobe PDF' format. Click here for file

Additional file 2 **The 'additional file **2**' is in Microsoft WORD format**. It includes two supplementary tables to make the context of the study clearer. Click here for file
