# Supplementary material for: Effects of caesarean section on maternal health in low risk nulliparous women: a prospective matched cohort study in Shanghai, China
Source: BMC Pregnancy Childbirth. 2010 Dec 2;10:78. doi: 10.1186/1471-2393-10-78 (PMC3014869; doi:10.1186/1471-2393-10-78)
Supplement: Additional file 2 — The 'additional file 2' is in Microsoft WORD format. It includes two supplementary tables to make the context of the study clearer. [file 1471-2393-10-78-S2.DOC]

**Supplementary Table S1: Additional Maternal characteristics by mode of delivery**

| **Characteristic** | **Caesarean section**  **N (%)** | **Vaginal delivery**  **N (%)** | **P value^*^** |
| --- | --- | --- | --- |
| Education |  |  | 0.2107 |
| Illiterate/elementary/middle school | 36 (11.96) | 50 (16.61) |  |
| High school | 157 (52.16) | 156 (51.83) |  |
| College and above | 108 (35.88) | 95 (31.56) |  |
| Occupation |  |  | 0.0895 |
| Factory | 160 (53.16) | 164 (54.49) |  |
| Civil service | 38 (12.62) | 28 ( 9.30) |  |
| Professional | 42 (13.95) | 29 ( 9.63) |  |
| Home care | 61 (20.27) | 80 (26.58) |  |
| Months of marriage |  |  | 0.2411 |
| ≤7 | 56 (18.60) | 62 (20.60) |  |
| 8-23 | 159 (52.82) | 171 (56.81) |  |
| ≥24 | 86 (28.57) | 68 (22.59) |  |
| Household monthly income per capita (RMB) |  |  | 0.0646 |
| <1000 | 48 (15.95) | 51 (16.94) |  |
| 1000-1999 | 107 (35.55) | 131 (43.52) |  |
| 2000-2999 | 67 (22.26) | 65 (21.59) |  |
| ≥3000 | 79 (26.25) | 54 (17.94) |  |

*Chi-square test

**Supplementary Table S2: Obstetric details by mode of delivery**

| **Characteristic** | **Caesarean section**  **N (%)** | **Vaginal delivery**  **N (%)** | **P value^*^** |
| --- | --- | --- | --- |
| Gestational age at birth (weeks)  *Median (Q_1_-Q_3_)* | 39 (39-40) | 39 (39-40) | 0.6422 |
| Premature rupture of membranes | 62 (20.60) | 79 (26.25) | 0.1018 |
| Analgesia for labor and delivery | 141 (46.84) | 125 (41.53) | 0.1891 |
| Postpartum analgesia | 156 (54.74) | 0 (0.00) | <0.001 |
| Extra hysterotonics given after delivery^$^ | 277 (95.85) | 230 (76.67) | <0.001 |
| Use of prophylactic antibiotics | 301 (100.00) | 145 (48.17) | <0.001 |
| Years of clinical experience (obstetrician / midwife) ^#^ |  |  | <0.001 |
| ≤5 | 8 ( 2.66) | 60 (19.93) |  |
| 6-15 | 204 (67.77) | 200 (66.45) |  |
| >15 | 89 (29.57) | 41 (13.62) |  |

* Statistical comparisons were made by Chi-square test, Fisher’s exact test or Wilcoxon rank sum test when appropriate.

$ Administration of extra oxytocin i.v. by continuous drip or other hysterotonics (such as carbetocin, prostaglandin PGE_2a_ or carboprost), in addition to the routine preventive single i.m. dose of oxytocin after delivery.

# Obstetrician for caesarean section and forceps delivery, midwife for spontaneous vaginal delivery.
